# The Effects of *Helicobacter pylori* Eradication on Proteinuria in Patients with Primary Glomerulonephritis

**DOI:** 10.1155/2014/180690

**Published:** 2014-02-24

**Authors:** Bahar Caliskan, Halil Yazici, Yasar Caliskan, Yasemin Ozluk, Mine Gulluoglu, Isin Kilicaslan, Aydin Turkmen, Mehmet Sukru Sever

**Affiliations:** ^1^Division of Pediatric Infectious Diseases, Department of Pediatrics, Istanbul Faculty of Medicine, Istanbul University, Istanbul, Turkey; ^2^Division of Nephrology, Department of Internal Medicine, Istanbul Faculty of Medicine, Istanbul University, Millet Caddesi, Capa, 34093 Istanbul, Turkey; ^3^Department of Pathology, Istanbul Faculty of Medicine, Istanbul University, Istanbul, Turkey

## Abstract

*Background*. Membranous nephropathy (MN) is a common cause of nephrotic syndrome. In most cases it is idiopathic, while it may also be secondary to many diseases. In this study, prevalence of *H. pylori* infection and the effects of *H. pylori* eradication on proteinuria levels were investigated. *Methods*. Thirty five patients with MN (19 male), 12 patients with IgA nephropathy (4 male) and 12 patients with focal segmental glomerulosclerosis (FSGS) (8 male) were studied. The presence of *H. pylori* antigen was investigated in renal tissues obtained by biopsy, and the effects of *H. pylori* eradication on proteinuria levels were investigated. *Results*. Immunohistochemistry with *H. pylori* antigen revealed no positive staining in the glomeruli of all patients. 19 patients (54%) with MN, 10 (83%) with IgA nephropathy and 4 (33%) with FSGS were positive for *H. pylori* stool antigen test (*P* = 0.045). Patients with *H. pylori* infection were administered eradication therapy (lansoprazole, 30 mg twice daily, plus amoxicillin, 0.75 g twice daily, plus clarithromycin, 250 mg twice daily, for 14 days). Before the eradication therapy the mean proteinuria of patients with MN, IgA nephropathy and FSGS were 2.42 ± 3.24 g/day, 2.12 ± 1.63 g/day and 1.80 ± 1.32 g/day, respectively. Three months after eradication, baseline proteinuria levels of patients with MN significantly decreased to 1.26 ± 1.73 g/day (*P* = 0.031). In all three groups there were no significant differences with regard to serum creatinine, albumin and C-reactive protein levels before and after eradication therapy. *Conclusions*. The eradication of *H. pylori* infection may be effective to reduce proteinuria in patients with MN, while spontaneous remission of MN could not be excluded in this patient cohort. This trial is registered with NCT00983034.

## 1. Introduction

Membranous nephropathy (MN) is a common cause of nephrotic syndrome (NS) in adults. In most cases it is idiopathic, while it may also be secondary to many diseases (e.g., infections, drugs, neoplasms, and autoimmune diseases) [1–5]. Histologically, MN is characterized by an accumulation of immune deposits on the outer aspect of the glomerular basement membrane. The immune deposits consist of IgG, often IgG4, so far unidentified antigens, and the membrane attack complex of complement C5b-9 [1, 6]. Functional impairment of the glomerulus causing proteinuria results from the formation of subepithelial immune deposits and complement activation [7, 8]. The key to a specific hypothesis-driven therapy is the understanding of the development of immune deposits, which first requires identification of the pathogenic antigen(s).

In 2009 Beck et al. identified the M-type phospholipase A2 receptor (PLA2R) as an important antigenic target [9]. The authors showed that PLA2R is expressed on podocytes and that antibodies against native PLA2R, primarily of the IgG4 subclass, were present in the serum of approximately 70% of patients with idiopathic MN [9]. The other antigens responsible for human MN have eluded identification, hepatitis B, hepatitis C, and *Helicobacter pylori *antigens; tumor antigens and thyroglobulin have also been detected in the subepithelial deposits, but there is no real proof that these antigens are pathogenic [1–5, 10, 11]. However, recent reports addressed the pathogenic role of *H. pylori* infection in MN [12, 13]. In these reports, remission of proteinuria was achieved in some patients following eradication of *H. pylori* infection [12, 13]. These reports might indicate that *H. pylori *infection might play an important role in the development of this disease. However, no controlled prospective study on this issue has been performed.

In this study, the prevalence of *H. pylori* infection and the effects of *H. pylori* eradication on the course of disease in patients with NS were investigated. This prospective study also compared the effects of *H. pylori* eradication on proteinuria levels in patients with MN versus other primary glomerulonephritides including IgA nephropathy and focal segmental glomerulosclerosis (FSGS). We also investigated the presence of *H. pylori* antigen in renal tissue from needle biopsy samples, using immunohistochemistry.

## 2. Materials and Methods

### 2.1. Patients

Fifty-nine patients (31 males and 28 females) with biopsy confirmed primary glomerulonephritis and persistent proteinuria, between ages of 18 and 70 [(mean age 44 ± 14 (range 19–69)], and being followed-up for more than six months were enrolled in this single center prospective study. Patients were grouped according to their diagnosis 1 month prior to the study. Thirty-five patients with MN (19 male, mean age: 46 ± 14 years), 12 patients with IgA nephropathy (4 male, mean age: 40 ± 13 years), and 12 patients with FSGS (8 male, mean age: 42 ± 15 years) were studied. All patients had been routinely screened for serum HBsAg, anti-HCV, antinuclear antibodies (ANA), anti-double-stranded DNA (anti-dsDNA), and complements. Inclusion criteria were biopsy-proven idiopathic MN, IgA nephropathy or FSGS, glomerular filtration rate (GFR) > 30 mL/min per 1.73 m^2^, persistent urinary protein excretion rate > 0.5 g/24 h over at least 6 months of full dose angiotensin converting enzyme inhibitors (ACEi) or angiotensin II receptor blockers (ARB) therapy, and no previous complete remissions or treatment with steroids or immunosuppressive drugs during the past year. Exclusion criteria were low (<30 mL/min/1.73 m^2^) glomerular filtration rate (GFR), diabetes mellitus, secondary glomerulonephritis, viral hepatitis, and history of gastric surgery. The patients who were on antibiotics, bismuth containing drugs, H2 receptor blockers, proton pump inhibitors, and antacids 3 months before the enrollment were excluded from the study.

Body mass index (BMI) was calculated by using the formula of weight/height^2^ (kg/m^2^). Blood pressure (BP) was measured in the sitting position after 5 min of rest with an ERKA sphygmomanometer (PMS Instruments Ltd., Berkshire, UK) with appropriately sized cuff on the right upper arm. Hypertension was defined as systolic BP > 140 mmHg or diastolic BP > 90 mmHg.

Fasting serum samples for biochemical studies were obtained between 8.00 and 8.30 in all cases. Laboratory values including complete blood cell count and serum levels of urea nitrogen (BUN), creatinine, total protein, albumin, total-, HDL-, LDL-, and VLDL-cholesterol, and triglycerides were measured by standard enzymatic procedures. High sensitive C-reactive protein (hs-CRP) levels were measured by nephelometric method (Dade Behring, GmbH, Marburg, Germany). GFR was predicted by using Chronic Kidney Disease Epidemiology Collaboration (CKD-EPI) equation [14]. Urinary protein-to-creatinine ratios (Up/c) were used to measure level of proteinuria.

Examinations of the patients confirmed to good medical and laboratory practices and the recommendations of the Declaration of Helsinki on Biomedical Research involving Human Subjects.

This study was approved by the Istanbul School of Medicine Clinical Studies Board.

### 2.2. Immunohistochemical Evaluation

All cases had adequate specimen for routine pathological examination, which included histochemical stains (haematoxylin and eosin, periodic acid-Schiff, methenamine silver-periodic acid, and Masson trichrome) and immunofluorescent stains (IgG, IgM, IgA, C1q, C3, C4, and fibrinogen). Renal biopsy specimens were fixed in Hollande's fixative, embedded in paraffin wax, and processed routinely. All histochemical and immunohistochemical stains were prepared using 3 micrometer paraffin sections. All cases were also surveyed for the *H. pylori* antigen in glomeruli using a standard immunohistochemical method. The monoclonal (Neomarkers, USA, dilution 1 : 1000, incubation 1 hour) and polyclonal (Mouse-anti-*Helicobacter pylori*, Dako, Denmark, 1 : 100 dilution, incubation 1 hour) antibodies were used for anti-*Helicobacter pylori* staining.

### 2.3. Detection of *H. pylori* Infection

Rapid HpSA test (Linear Chemicals, Barcelona, Spain) was performed in fresh stool according to the manufacturer's recommendations without knowledge of the *H. pylori* status by a single biologist. Rapid HpSA test provides a simple alternative to the evaluation of gastric biopsy specimens for *H. pylori* and the value of test for the diagnosis of *H. pylori* infection is well documented in the previous reports [15, 16]. This test is a rapid 10-minute test, qualitative via a lateral flow chromatography technique. It is simple to perform and has possible advantages over other noninvasive tests, detecting actual antigen indicating current active infection [17]. The test was considered negative if only blue colored band (control line) appeared across the white central area of the reaction strip and positive when, in addition to the control line, a distinguishable pinkish red band (test line) also appeared across the central zone of the reaction strip. Any line or color appearing after 10 min has no diagnostic value. Tests were also considered invalid if the control band was absent.

### 2.4. *H. pylori* Eradication Therapy

Proton pump inhibitor-based triple therapy was administered to patients whose Rapid HpSA test was found positive. The therapy protocol was as follows: lansoprazole, 30 mg twice daily, plus amoxicillin, 0.75 g twice daily, plus clarithromycin, 250 mg twice daily, for 14 days. Serum creatinine, total cholesterol, albumin, hs-CRP and daily proteinuria levels, and GFRs were assessed before and after the eradication therapy. The efficacy of *H. pylori* eradication was evaluated by a validated Rapid HpSA test 3 months after completion of therapy.

### 2.5. Study Protocol

Renal biopsy specimens were surveyed for the *H. pylori* antigen in glomeruli using an immunohistochemical method. Baseline complete blood cell count and serum levels of BUN, creatinine, total protein, albumin, total and LDL-cholesterol, triglycerides and hs-CRP, Up/c, and GFR values were measured. Rapid HpSA test (Linear Chemicals, Barcelona, Spain) was performed in all patients. Patients with *H. pylori* infection were administered eradication therapy (lansoprazole, 30 mg twice daily, plus amoxicillin, 0.75 g twice daily, plus clarithromycin, 250 mg twice daily, for 14 days). Eradication therapy was stopped and patients were followed for an additional three months. At the end of the follow-up period, Rapid HpSA test, serum creatinine, total protein, albumin, total and LDL-cholesterol, triglycerides, hs-CRP levels, Up/c, and GFR values were assessed again.

### 2.6. Statistical Analysis

The statistical analysis was carried out by Statistical Package for Social Sciences for Windows version 15.0 (SPSS Inc., Chicago, IL, USA). Continuous variables were expressed as mean ± SD. Parametric and nonparametric tests were used according to the distribution pattern of the data of each variable. Associations between groups and dichotomous variables were examined by using the chi-square test or Fisher's exact test where appropriate. Statistical comparisons of individual groups were based on Student's *t*-test for continuous variables and on Fisher's exact test for discrete variables. Paired *t*-test was used to compare clinical and laboratory features before and after conversion. Nonparametric methods were also performed using the Wilcoxon signed rank test for differences within groups (paired data) or the Mann-Whitney *U* test for analysis between groups (unpaired data). All statistical tests performed were two sided and the level of significance was 0.05.

## 3. Results

The baseline clinical and laboratory features of the patients are shown in Table 1. There were no differences regarding age, gender, smoking status, systolic/diastolic BP, hemoglobin, glucose, serum creatinine, total cholesterol, LDL cholesterol, triglyceride, total protein, albumin, hs-CRP, GFR, and Up/c levels among the three groups (Table 1).

Immunohistochemistry for *H. pylori* antigen revealed no positive staining in the glomeruli from renal biopsy specimens in all patients. 19 patients (54%) with MN, 10 patients (83%) with IgA nephropathy, and 4 patients (33%) with FSGS were positive for *H. pylori* stool antigen test (*P* = 0.045). The baseline clinical and laboratory features of the patients who were positive for *H. pylori* stool antigen test are shown in Table 2. There were no differences regarding age, gender, smoking status, systolic/diastolic BP, hemoglobin, glucose, total cholesterol, LDL cholesterol, triglyceride, total protein, albumin, hs-CRP, Up/c, and GFR levels among the three groups (Table 2). The mean serum creatinine level of patients with MN (0.98 ± 0.29 mg/dL) was significantly lower than the patients in the other groups (*P* = 0.011). Patients with *H. pylori* infection were administered eradication therapy (lansoprazole, 30 mg twice daily, plus amoxicillin, 0.75 g twice daily, plus clarithromycin, 250 mg twice daily, for 14 days). All patients were receiving angiotensin converting enzyme inhibitors or angiotensin II receptor blockers since 6 months before the study entry. All patients had received immunosuppressive therapy including steroid, cyclosporine A, mycophenolate mofetil, and azathioprine and there was no need to change the treatment during the study period.

Eradication was successful in all patients. No significant side effect was observed in any patient during the eradication therapy. Laboratory features of patients before and after *H. pylori* eradication therapy are shown in Table 2. Three months after eradication therapy, there were no differences regarding systolic/diastolic BP, hemoglobin, serum glucose, creatinine, total protein, albumin, total cholesterol, LDL cholesterol, triglyceride, hs-CRP, and Up/c levels among the three groups (Table 2).

Before the eradication therapy, the mean urinary protein excretion of patients with MN, IgA nephropathy, and FSGS was 2.42 ± 3.24 g/day, 2.12 ± 1.63 g/day, and 1.80 ± 1.32 g/day, respectively. Three months after eradication therapy, baseline proteinuria levels of patients with MN significantly decreased to 1.26 ± 1.73 g/day (*P* = 0.031). Decrease was also noted in patients with IgA nephropathy (1.58 ± 1.59 g/day; *P* = 0.13), however without reaching statistical significance. In FSGS group baseline proteinuria levels (1.80 ± 1.32 g/day) increased to 1.91 ± 1.21 g/day, but the difference was not statistically significant (*P* = 0.91). In all three groups after the eradication therapy there were no significant differences with regard to hemoglobin, serum creatinine, total protein, albumin, total cholesterol, LDL cholesterol and hs-CRP levels compared to baseline values. GFR values after the eradication therapy were also similar to values before the therapy.

## 4. Discussion

In the present study, the eradication of *H. pylori* infection successfully reduced proteinuria in patients with MN. To our knowledge, this is the first study to show that the eradication of *H. pylori* infection has an ameliorating effect on urinary protein excretion.

Since the first report in 1997 in which Nagashima et al. have reported the presence of *H. pylori* antigens in the glomeruli of MN patients [11], few reports have been published on the association between *H. pylori* and MN [10–13]. In particular, a higher prevalence of *H. pylori* infection in patients affected by MN has been described and there is growing evidence for an association between *H. pylori* and MN. A recent study showed a significantly higher *H. pylori* infection rate (66%) in MN patients than in their control group (44%) consisting of normal healthy subjects [12]. In the present study, the prevalence of *H. pylori *infection was lower in patients with MN (54%) than in patients with IgA nephropathy (83%). Additionally, the prevalence of *H. pylori* infection was similar between patients with MN and FSGS. Barratt et al. also reported a high incidence of *H. pylori* seropositivity in patients with IgA nephropathy and suggested a possible explanation for this high incidence of *H. pylori* seropositivity [18]. As suggested by this study, in IgA nephropathy, there is an increase in gastric permeability resulting in excessive *H. pylori* antigenemia and systemic immune activation which may cause high incidence of *H. pylori* seropositivity [18]. However, the explanation for this high seropositivity rate is still unclear. Although previous studies may suggest that MN patients are prone to *H. pylori* infection, it is possible that hypoalbuminemia or hypoglobulinemia (in the case of NS) may predispose patients to becoming immunocompromised and may lead to *H. pylori* infection [12]. However, in the present study, the serum albumin levels of patients with MN and IgA nephropathy were similar.

Another interesting finding of our study is that, although previous studies detected *H. pylori* antigens in the glomerulus of renal biopsy specimens from patients with MN, the immunohistochemistry findings in the present study revealed no deposition of *H. pylori*. These renal biopsies performed at the early stage of the MN for the histological diagnosis; thus, there is a possibility that enough amounts of *H. pylori* antigens, which can be detected by immunohistochemistry might not have deposited in our patients' glomeruli. However, there were also biopsy specimens showing stage 2 or 3 MN; thus, this hypothesis does not seem likely. On the other hand, there is another possibility that instead of *H. pylori *antigen, anti-*H. pylori* antibodies might be reacting with podocyte antigens to form immune deposits in situ under the subepithelium, resulting in the development of MN. If this is true, all MNs in the present study are supposed to be secondary MN, which can be demonstrated by the negative staining of IgG4 and PLA2R1. Although no definitive data are available on the pathogenesis of these phenomena, previous studies showed that antibodies against *H. pylori* may react with extragastric tissues, such as glomerular capillary walls, ductal cells of the salivary gland, and renal tubular cells [11, 19]. However, the mechanisms behind this phenomenon are still unclear.

Previous case reports reported that eradication of *H. pylori* markedly reduced urinary protein excretions in four of five MN patients who were also receiving corticosteroid therapy [12, 13]. The effects of *H. pylori* eradication on proteinuria levels in patients with MN versus other primary glomerulonephritides including IgA nephropathy and FSGS were assessed for the first time, in this cohort study. Three months after the eradication therapy, significant decrease in proteinuria levels and increase in serum albumin levels were noted in patients with MN. Decrease in proteinuria levels was also noted in patients with IgA nephropathy, however without reaching statistical significance. Patients with IgA nephropathy, FSGS, and MN may response differently to the therapy in terms of alleviation of proteinuria. However, these results also suggest that eradication of *H. pylori* infection has an ameliorating effect on urinary protein excretion in the course of MN. In other words, *H. pylori *infection may be a secondary etiological factor in MN and the eradication of this infection might be an affirmative therapy for MN patients with* H. pylori *infection. However, spontaneous remission of MN could not be excluded in this patient cohort. Previous studies reported that 10% of nontreated patients with MN underwent spontaneous remission at 12 months, 16% at 24 months, and 22% at 36 months [20]. Therefore, the reduction of proteinuria in the present study might either be the consequence of eradication therapy or represent spontaneous remission of MN. In order to minimize the effect of spontaneous remission on the results, the patient cohort was enrolled in this study after a six-month period of follow-up.

In conclusion, the eradication of *H. pylori* infection successfully reduced proteinuria in patients with MN, while spontaneous remission of MN could not be excluded in this patient cohort. Further well designed in vitro, epidemiological, and controlled intervention studies are needed in order to identify whether and by which molecular mechanisms *H. pylori* may play role in MN pathogenesis.

## Figures and Tables

**Table 1:** The baseline demographic, clinical, and laboratory features of the study patients.

	MN group (*n* = 35)	IgAN group (*n* = 12)	FSGS group (*n* = 12)	*P* value
Male/female	19/16	4/8	8/4	NS
Age (years)	46 ± 14	40 ± 13	42 ± 15	NS
BMI (kg/m^2^)	24.9 ± 4.3	27.7 ± 5.7	25.8 ± 3.6	NS
Systolic BP (mmHg)	121 ± 20	126 ± 18	123 ± 18	NS
Diastolic BP (mmHg)	75 ± 12	81 ± 16	79 ± 13	NS
Hemoglobin (g/dL)	12.9 ± 2.0	12.3 ± 1.0	12.3 ± 1.9	NS
Creatinine (mg/dL)	0.98 ± 0.31	1.14 ± 0.40	1.26 ± 0.42	NS
Albumin (g/dL)	3.53 ± 0.82	3.85 ± 0.79	3.70 ± 0.44	NS
T. cholesterol (mg/dL)	213 ± 0.53	225 ± 57	261 ± 142	NS
Triglyceride (mg/dL)	177 ± 128	188 ± 126	214 ± 115	NS
GFR (mL/min)	87 ± 29	72 ± 25	72 ± 30	NS
Proteinuria (g/day)	2.76 ± 3.10	2.04 ± 1.60	3.55 ± 2.69	NS

AMN: membranous nephropathy, IgAN: IgA nephropathy, FSGS: focal segmental glomerulosclerosis, NS: not significant, GFR: glomerular filtration rate.

**Table 2:** Clinical and laboratory features of the patients before and after *H. pylori* eradication therapy.

	MN group (*n* = 19)	IgAN group (*n* = 10)	FSGS group (*n* = 4)	*P* value
Male/female	13/6	4/6	3/1	NS
Age (years)	45 ± 13	39 ± 12	37 ± 11	NS
BMI (kg/m^2^)	25.0 ± 4.7	27.9 ± 5.8	25.4 ± 3.5	NS
Before therapy				
Systolic BP (mmHg)	119 ± 22	133 ± 18	123 ± 8	NS
Diastolic BP (mmHg)	76 ± 13	78 ± 10	83 ± 10	NS
Hemoglobin (g/dL)	13.1 ± 2.0	12.3 ± 1.1	12.5 ± 0.9	NS
Glucose (mg/dL)	92 ± 13	87 ± 13	92 ± 14	NS
Creatinine (mg/dL)	0.98 ± 0.29	1.21 ± 0.41	1.60 ± 0.50	0.011
T. protein	6.38 ± 1.11	6.52 ± 1.35	6.30 ± 0.70	NS
Albumin (g/dL)	3.66 ± 0.75	3.76 ± 0.83	3.90 ± 0.32	NS
Proteinuria (Up/C) (g/day)	2.42 ± 3.24	2.12 ± 1.63	1.80 ± 1.32	NS
GFR (mL/min)	89 ± 29	69 ± 27	56 ± 20	NS
T. cholesterol (mg/dL)	214 ± 54	232 ± 60	235 ± 82	NS
Triglyceride (mg/dL)	179 ± 149	196 ± 138	223 ± 165	NS
hs-CRP	2.66 ± 3.20	4.10 ± 3.72	7.15 ± 8.28	NS
After therapy				
Systolic BP (mmHg)	116 ± 22	135 ± 16	119 ± 9	NS
Diastolic BP (mmHg)	74 ± 13	85 ± 12	80 ± 8	NS
Hemoglobin (mg/dL)	12.9 ± 1.8	12.2 ± 1.3	12.7 ± 1.2	NS
Creatinine (mg/dL)	1.01 ± 0.32	1.15 ± 0.42	1.78 ± 0.59	0.005
T. protein (g/dL)	6.66 ± 0.92	6.06 ± 1.31	6.73 ± 0.31	NS
Albumin (g/dL)	3.89 ± 0.61	3.61 ± 0.91	4.06 ± 0.38	NS
Proteinuria (g/day)	1.26 ± 1.73	1.58 ± 1.59	1.91 ± 1.21	NS
GFR (mL/min)	87 ± 29	72 ± 30	51 ± 24	NS

AMN: membranous nephropathy, IgAN: IgA nephropathy, FSGS: focal segmental glomerulosclerosis, NS: not significant, GFR: glomerular filtration rate.
